# Release factor 3 degradation enhances aminoglycoside-mediated translational readthrough at *MECP2* nonsense mutations

**DOI:** 10.1007/s00109-026-02728-z

**Published:** 2026-10-22

**Authors:** Dennis Lebeda, Daniela Smoljanow, Julia Hofhuis, Sven Thoms

**Affiliations:** Department for Biochemistry and Molecular Medicine, Medical School OWL, Bielefeld University, Bielefeld, Germany

**Keywords:** Rett syndrome, Translational readthrough, Rare disease, *MeCP2*, eRF3, Aminoglycoside

## Abstract

Premature termination codons (PTCs) in the *MECP2* gene are a major cause of Rett syndrome, resulting in truncated proteins and loss of function. Pharmacological induction of translational readthrough (TR) can restore full-length protein, but clinical translation is hindered by the limited efficiency and toxicity of current inducers. Here, we investigated whether targeted degradation of the eukaryotic Release Factor 3a by the small molecules CC-885 or CC-90009 enhances TR at *MECP2* PTCs in HeLa cells. Using a dual-fluorescence reporter assay, we found that neither degrader alone significantly increased TR efficiency. However, cotreatment with the TR inducer G418 produced a synergistic effect, resulting in increased TR compared to G418 alone. Although gentamicin, a less toxic TR inducer, led to only weak TR induction on its own, combination with CC-885 resulted in TR levels similar to G418. Combined CC-885 and G418 treatment enabled recovery of full-length MeCP2 protein as well as proper nuclear localization, consistent with functional recovery of the nuclear localization signal. These findings establish eRF3a degradation as a potential strategy to enhance TR and suggest that the combination of aminoglycosides with release factor degraders could provide a less toxic yet effective therapeutic option for PTC-associated disorders.

## Key Messages

• In combination with aminoglycosides, eRF3 degrader CC-885 enhances translational readthrough, demonstrating its role as a potentiator.

• eRF3 degradation alone is insufficient to trigger translational readthrough.

• Translational readthrough efficiency is strongly dependent on stop codon and context identity.

• Co-treatment with CC-885 and aminoglycosides allows reduction of aminoglycoside dose to achieve relevant readthrough efficiencies.

• Co-treatment restores full-length MeCP2 protein expression and promotes nuclear localization of the recovered protein.

## Introduction

Mutations in the *MECP2* gene are the primary cause of Rett syndrome, a severe X-linked neurodevelopmental disorder that primarily affects females. The disease is characterized by an early onset between 6 and 18 months of age after normal development, followed by developmental regression, loss of acquired motor and communication skills, seizures, autistic features, and a range of autonomic dysfunctions [1, 2].

*MECP2* encodes the methyl-CpG-binding protein 2 (MeCP2), a transcription factor that binds methylated DNA via its methyl-CpG-binding domain and recruits factors associated with transcriptional repression and activation [2–5]. MeCP2 exhibits two major isoforms, e1 and e2, which differ in their start codon, with e1 being the predominant form in the brain [6]. Two nuclear localization signal (NLS) domains are present in MeCP2, but the presence of only the upstream NLS appears to be sufficient for nuclear targeting [7]. A large proportion (35%) of the pathogenic *MECP2* mutations are nonsense mutations [2]. These introduce premature termination codons (PTCs) that result in truncated, non-functional proteins. Alternatively, PTCs may trigger nonsense-mediated mRNA decay (NMD), further reducing expression of the functional protein [8]. Canonical NMD is triggered by PTCs located upstream of the last exon-exon junction. Among RTT-causing *MECP2* PTCs, p.S68X lies upstream of the last splice site (approximately amino acid position 138; NM_001110792.2) and is therefore subject to NMD, whereas the majority of RTT PTCs, including those investigated in this study, reside within the terminal exon and consequently escape canonical NMD. This NMD escape results in stable mRNA, which represents a viable template for TR-mediated restoration of full-length protein. Translational readthrough (TR) refers to the process by which ribosomes recode stop codons by incorporating near-cognate tRNAs, enabling peptide chain elongation and thus synthesis of the full-length protein. Endogenous TR is usually rather uncommon, with a probability of < 0.1%. However, some transcripts are regulated by high TR efficiencies to provide an evolutionarily conserved mechanism in which ribosomes read through a stop codon to produce a C-terminally extended protein isoform with distinct functional or subcellular localization properties [9–11]. Further, TR can be induced pharmacologically and represents a promising therapeutic approach to bypass PTCs in corresponding diseases [12–14]. The efficiency of TR induction is strongly influenced by the stop codon context (SCC), which is defined by the stop codon type—UAA, UAG or UGA—and the surrounding nucleotides. UGA is the most reathrough-prone stop codon and UAA is the least susceptible to TR induction. A cytosine immediately downstream of a stop codon further enhances TR inducibility [9, 15–17].

Among TR inducers, the aminoglycoside G418 (geneticin) shows a high level of TR induction and is therefore well suited to experimentally analyze TR in a preclinical setting. G418 binds to the ribosomal decoding center, leading to destabilization of codon-anticodon interactions at stop codons and subsequent near-cognate tRNA incorporation [18]. However, its high cytotoxicity and poor safety profile preclude clinical application [12, 19]. The aminoglycoside gentamicin is less toxic but generally induces lower TR efficiency [20].

Pharmacological TR has been investigated at *MeCP2* PTCs for cellular models, animal models, and patient-derived cells [7, 21–25]. However, these studies mostly covered the four most occurring PTCs (p.R168X, p.R255X, p.R270X, p.R294X), and high doses of aminoglycosides are necessary to induce TR at *MECP2* PTCs. Further, these studies lack a cytotoxicity readout to evaluate MeCP2 recovery versus G418 toxicity. A novel, mechanistically different approach to boost TR is the degradation of eukaryotic release factor 3a (eRF3a), a GTPase crucial for translation termination. Small molecules like CC-885 and CC-90009 function as cereblon modulators, which act as molecular glues, recruiting the cereblon substrate receptor of the CRL4 E3 ubiquitin ligase complex to eRF3a as a neo-substrate, thereby inducing its ubiquitination and subsequent proteasomal degradation [26]. This approach impairs termination while raising the likelihood of TR, which has been shown in reporter systems [27–29]. Its potential to increase TR induction levels might allow the reduction of the effective dose of highly toxic aminoglycosides to non-toxic levels or the potentiation of less toxic agents that are otherwise only weak inducers.

In this study, we tested two central hypotheses. First, we asked whether pharmacological degradation of eRF3a by CC-885 boosts aminoglycoside-induced TR at several *MECP2* PTCs. Second, we examined whether this potentiation allows either enhancement of weak TR inducers such as gentamicin or a reduction of the effective G418 concentration.

Using several experimental setups to investigate TR efficiency – by using a reporter model, assessing full-length MeCP2 recovery, and analysing its subcellular localization - we demonstrate that eRF3a degradation alone does not trigger TR but robustly amplifies aminoglycoside-dependent TR in a SCC-dependent manner. Importantly, this synergy allows a substantial reduction of the G418 dose while preserving recovery of full-length MeCP2 and its nuclear localization.

## Results

### eRF3a degradation enhances aminoglycoside-induced readthrough across *MECP2* nonsense mutations

**Fig. 1:**
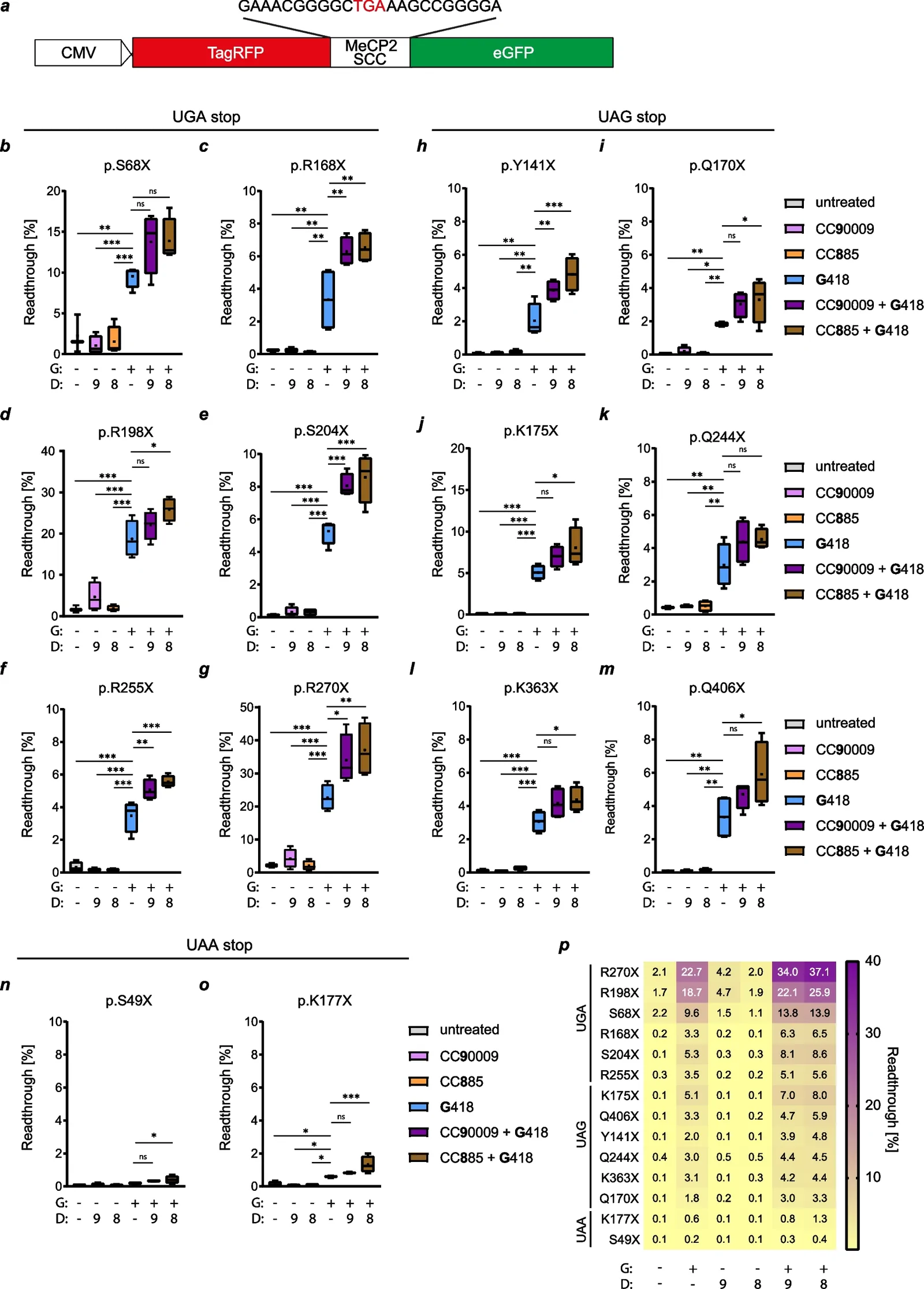
MeCP2 PTC screening for synergistic TR induction upon G418 and eRF3a degrader treatment. **a** Schematic representation of the dual reporter construct used for TR quantification. 23-nucleotides MeCP2 PTC contexts are flanked by an N-terminal RFP and a C-terminal GFP. **b-o ** TR efficacy of 14 different MeCP2 nonsense mutations in reporter-transfected cells. Cells were left untreated or treated for 24 h with G418 (G) [100 ng/µL], eRF3a degrader (D) CC-90009 (9) [30 nM], and eRF3a degrader CC-885 (8) [1 nM] alone or in a combination of G418 and either eRF3a degrader. Six MeCP2 PTCs were coded UGA (**b-g**), six were UAG (**h-m**), and two were UAA (**n**–**o**). TR was calculated as GFP/RFP fluorescence ratio normalized to a 100% readthrough control. Bar = median, dots = mean, error bar = SD from n = 4 independent experiments. Statistical significance was determined by two-way ANOVA compared to G418 treatment with Bonferroni’s post hoc test (* *p* < 0.05, ** *p* < 0.01, *** *p* < 0.001). **p** Heatmap of all measurements sorted from highest to lowest mean TR after CC-885 and G418 treatment

To analyze the general applicability of eRF3a degradation for nonsense mutations in *MECP2*, we employed a dual reporter assay covering 14 clinically relevant PTCs. Each PTC and its surrounding 20 nucleotides separated an upstream RFP and a downstream GFP coding sequence. Inducing TR leads to expression of the GFP signal that can be detected and quantified by flow cytometry (Fig. 1a). We used 100 ng/µL G418 for TR induction and either 1 nM CC-885 or 30 nM CC-90009 to promote targeted degradation of eRF3a.

As expected, G418-mediated TR efficiencies strongly depended on the SCC, with UGA being the most permissive, ranging between approximately 3% for p.R255X and 24% for p.R270X (Fig. 1b-g). UAG PTCs show intermediate responses upon G418 induction, reaching TR efficiencies between 2 and 5% (Fig. 1h-m), and UAA was least permissive, with under 1% (Fig. 1n-o). Generally, treatment with G418 significantly induced TR at almost all PTCs except p.S49X (UAA stop), but TR efficiencies remained modest or low in several variants.

Combination with either eRF3a degrader increased G418-induced TR at most PTCs. CC-885 displayed equal or increased G418-induced TR at a ~ 30-fold lower concentration and with broader statistical significance across PTCs compared to G418 alone. We therefore focused on the use of CC-885 in this study. PTCs that already showed substantial G418-dependent readthrough, such as p.R198X (19%) and p.R270X (23%), exhibited the most pronounced increases upon co-treatment with CC-885 to 26% and 37%, respectively (Fig. 1d,g). The heatmap summarizing all tested PTCs underscores the capacity of eRF3a degradation to enhance aminoglycoside-induced readthrough (Fig. 1p). In contrast, UAA PTCs - characterized by low basal inducibility - displayed only marginal increases despite eRF3a degradation. Importantly, eRF3a degrader treatment alone failed to induce statistically significant TR at any tested PTC, indicating that eRF3a depletion does not initiate TR on its own but amplifies ongoing TR events. RFP intensity remained stable under eRF3a degrader treatment alone but was reduced upon G418 treatment, both alone and in combination, which is consistent with aminoglycoside-mediated effects on translational fidelity. GFP intensities increased upon G418 and degrader co-treatment, consistent with the TR efficiencies reported in Fig. 1, while degrader treatment alone had no substantial effect on GFP signal (Suppl. Figure 1). Together, these findings establish eRF3a degradation as a TR potentiating mechanism rather than a primary inducing stimulus.

### CC-885 reduces eRF3a protein levels and displays dose-dependent effects on cell viability

**Fig. 2:**
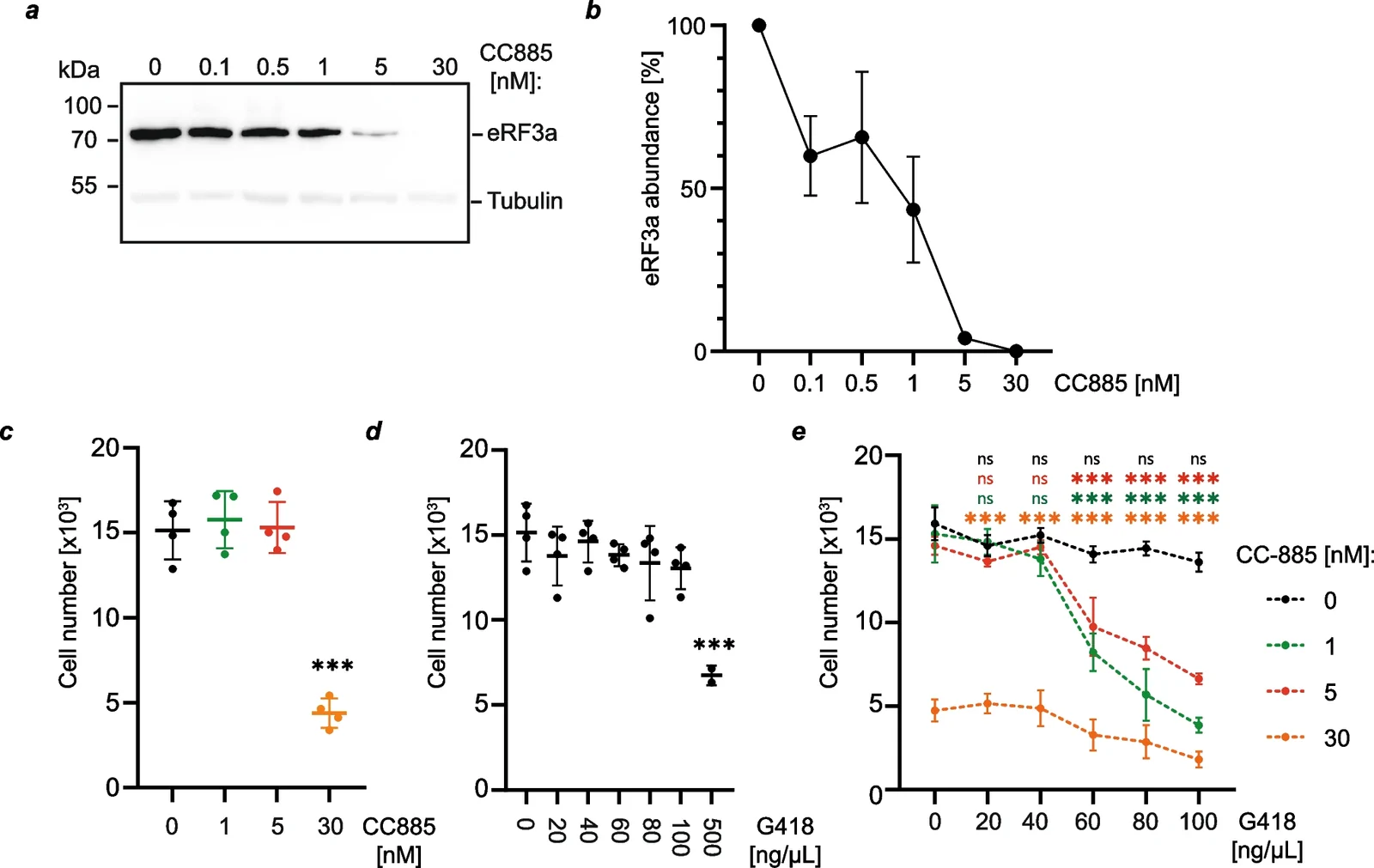
Effect of CC-885 on cellular eRF3a content and cell viability in combination with G418. **a** eRF3a expression after a 24-h treatment with the indicated concentrations of CC-885. **b** Densitometric quantification of western blot bands. The eRF3a signal was normalized to Tubulin signals and compared to untreated controls. *n* = 3 individual western blots. Error bars = SD. **c-e** Cell viability assays of HeLa cells that were treated with the indicated concentrations of either G418 (**c**), CC85 (**d**), or in combination (**e**). Cell viability was assessed after 24 h using the OrangU assay. Bar = mean, error bar = SD from n = 3. Cell number was calculated from a standard curve. Statistical significance was determined by one-way ANOVA (**c-d**) or two-way ANOVA (**e**) with Bonferroni’s post hoc test (* *p* < 0.05, ** *p* < 0.01, *** *p* < 0.001)

To confirm the eRF3a-degrading activity of the compound CC-885, we analyzed eRF3a expression after G418 and CC-885 treatment in HeLa cells at various concentrations (Fig. 2a-b). In line with their reported mechanism of action, CC-885 induced a marked concentration-dependent degradation of eRF3a in the low nanomolar range. For 1 nM and 5 nM, 50% and 95% of the intracellular eRF3a level is degraded, respectively. Using 30 nM, the eRF3a protein is not detectable anymore, indicating complete eRF3a degradation. To assess CC-885-induced toxicity, we analyzed the HeLa cell viability after 24 h of treatment with CC-885 and G418, alone or in combination.

CC-885 alone did not affect cell viability at concentrations up to 5 nM, whereas viability was markedly reduced at 30 nM (Fig. 2c). G418 also exhibited dose-dependent cytotoxicity, consistent with its known disturbance of translation fidelity. As both pathways - eRF3a degradation by CC-885 and ribosomal disturbance by G418 - are capable of inducing cytotoxicity, we next examined their combined influence on cell survival. Notably, increased cytotoxicity was observed when CC-885 (≥ 1 nM) was combined with G418 at concentrations ≥ 60 ng/µL (Fig. 2e). Individually, both compounds were well tolerated by HeLa cells at these concentrations, indicating that the convergence of these orthogonal mechanisms increases cytotoxicity. We found that a combination of 40 ng/µL G418 and 5 nM CC-885 is the tolerable application limit for HeLa cells that does not impair cell viability under these experimental conditions.

### Degradation of eRF3a allows reduction of aminoglycoside concentration and boosts weak translational readthrough inducers at selected *MECP2* PTCs

Using the dual reporter assay, we next examined four representative PTCs (Fig. 1a) in greater detail: p.R198X (UGA), p.S68X (UGA), p.K175X (UAG), and p.K177X (UAA). These mutations were selected to represent distinct stop codon types and experimental rationales: p.R198X contains a UGA stop codon in a highly permissive sequence context, exemplifying high TR accessibility within RTT-associated PTCs; p.S68X was chosen because the truncated protein lacks the nuclear localization signal, providing an unambiguous functional readout for successful TR via nuclear translocation; p.K175X (UAG) and p.K177X (UAA) were included as mechanistic contrasts to analyze the SCC-dependence of the combinatorial effect. The primary objective of this mutation selection was to characterize the mechanistic basis of eRF3a degradation-mediated TR potentiation rather than to reflect clinical prevalence. Both p.R198X and p.S68X showed high levels of induction in the initial screen (Fig. 1b,d). However, while p.R198X responded strongly to the combination of G418 and CC-885, p.S68X did not display a significant increase compared to G418 alone. To capture the effects across all stop codon types, we included p.K175X and p.K177X as representatives of UAG and UAA contexts, respectively.

**Fig. 3:**
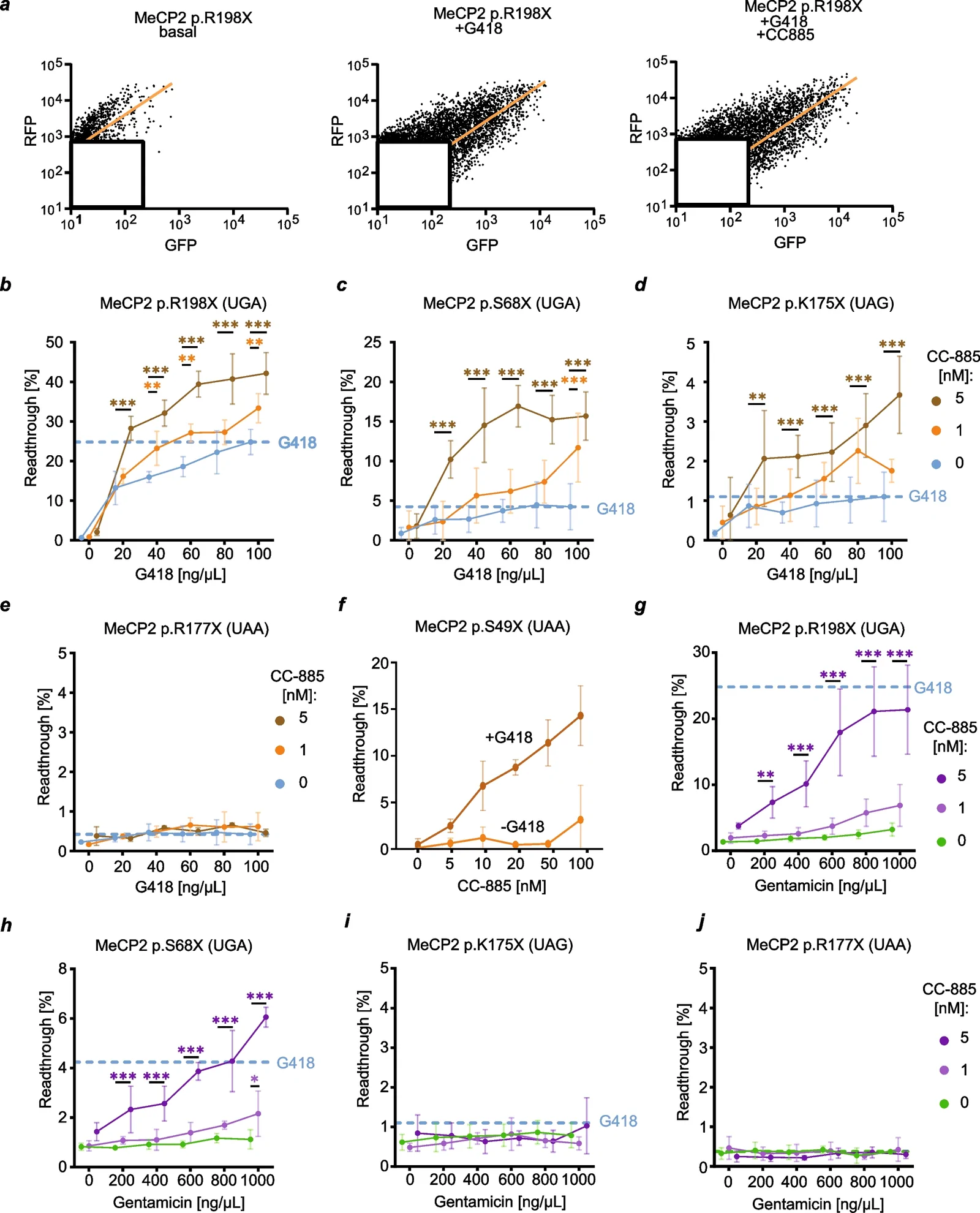
Concentration-dependent CC-885 synergy with aminoglycosides to enhance translational readthrough at *MECP2* PTCs. Dual reporter assay in cells transfected with RFP-MeCP2 SCC-GFP constructs used to measure TR at two TGA stops, one TAG stop and one TAA stop. Cells were treated with G418 in the presence or absence of CC-885 for 24 h. TR efficiency was calculated as GFP/RFP fluorescence ratio normalized to a 100% readthrough control. **a ** Representative dot plot visualizing GFP vs RFP of MeCP2 p.R198X, untreated or treated with G418 alone or in combination with CC-885. Orange line shows linear fit. **b-i** Transfected cells treated with G418 or gentamicin in the presence or absence of CC-885. Blue dotted line indicates TR value at 100 ng/µL G418 for each construct. Data represent mean ± SD from n = 3 independent experiments. Statistics: two-way ANOVA to control G418 group (single treatment) with Bonferroni correction (* *p* < 0.05, ** *p* < 0.01, *** *p* < 0.001). **f** TR efficiency of the *MECP2* p.S49X SCC treated by CC-885 or in combination with G418 [100ng/µL]

We next assessed TR efficiencies over a range of G418 and CC-885 concentrations. This allowed us to determine whether eRF3a degradation can potentiate TR induction, particularly at lower, potentially less toxic G418 concentrations. CC-885 significantly enhanced G418-induced TR at the UGA and UAG PTCs, confirming the synergistic effect observed in the broader screen (Fig. 3b-d). Using 1 nM of CC-885 with G418, the TR efficiency was boosted to only a small extent compared to the corresponding G418 treatment alone. However, increasing the CC-885 concentration to 5 nM markedly potentiated TR at UGA and UAG stop codons. The lowest investigated G418 concentration at 20 ng/µL surpassed the effect of a five-fold increased G418 concentration when combined with 5 nM of CC-885. A concentration-dependent effect further boosted TR efficiency up to 40% at the p.R198X PTC (Fig. 3b). Thus, the application limit of 40 ng/µL G418 with 5 nM CC-885 significantly outperformed TR levels of 100 ng/µL G418 alone (p.R198X: 25% to 31%; p.S68X: 4.5% to 15%; p.K175X: 1.1% to 3.5%) (Fig. 3b-d). By contrast, the p.K177X variant (UAA) remained unresponsive to TR induction under all tested conditions (Fig. 3e). Using a heavily increased concentration of CC-885 at cytotoxic levels, concentration-dependent TR induction was observed at another UAA PTC (p.S49X) when combined with G418 (Fig. 3f). Interestingly, CC-885 concentrations over 30 nM still show concentration-dependent TR induction rates at p.S49X, although the intracellular eRF3a content is already decreased to a non-detectable level at 30 nM.

Next, we tested the ability of CC-885-mediated TR potentiation upon gentamicin treatment. Gentamicin is a less toxic aminoglycoside, but exhibits a lower ability to induce TR than G418. At all investigated PTCs, gentamicin treatment alone led to almost no TR induction at a tenfold higher concentration than G418 (Fig. 3g-j). However, at UGA stop codons, the combination with 5 nM CC-885 significantly increased TR efficiencies to 20% at p.R198X (Fig. 3g) and 6% at p.S68X (Fig. 3h), which surpass G418 treatment alone. The combination did not induce TR at p.K175X (UAG) and p.K177X (UAA) (Fig. 3i-j).

These findings demonstrate that eRF3a degradation potentiates aminoglycoside-induced TR and provides a flexible strategy to enhance weak TR inducers on the one hand and reduce the concentration of aminoglycosides on the other hand. However, this dual treatment approach is restricted to permissive SCCs.

### Restoration of full-length MeCP2 protein by the co-treatment of aminoglycosides and eRF3a degradation

**Fig. 4:**
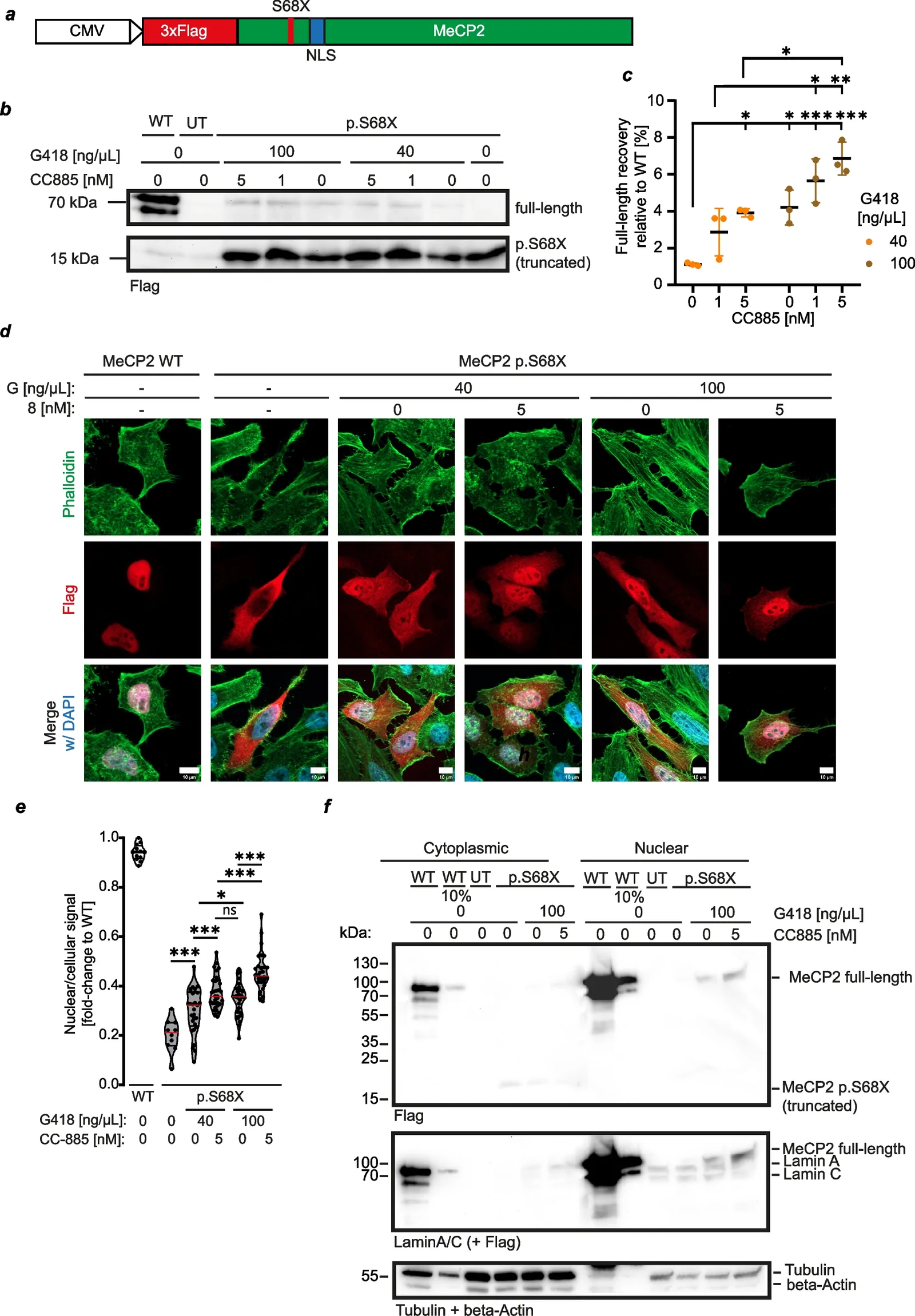
Nuclear localization of recovered full-length MeCP2. **a** 3xFlag-MeCP2 WT or p.S68X constructs used for transfection. The p.S68X PTC leads to expression of truncated MeCP2 lacking a nuclear localization signal (NLS, blue). **b** Expression of 3 × FLAG-tagged MeCP2 WT and PTC variant p.S68X. Transfected cells were treated for 24 h with G418 alone or in combination with CC-885. Full-length and truncated MeCP2 variants were detected using anti-FLAG antibodies; truncated MeCP2 served both as a transfection and loading control. WT = Wildtype, UT = Untransfected **c** Densitometric quantification of full-length MeCP2 normalized to MeCP2 WT (mean ± SD, n = 3). Statistical significance assessed by two-way ANOVA with Bonferroni’s post hoc test (* *p* < 0.05, ** *p* < 0.01, *** *p* < 0.001). **d** Immunofluorescence of transfected cells left untreated (-) or treated with G418 alone or in combination with CC-885. Z-stack projections of Phalloidin-Alexa 488 (green), 3xFLAG-MeCP2 (red) and DAPI (blue) show increased nuclear accumulation of MeCP2 after dual treatment. WT = Wildtype, UT = Untransfected. White scale bars = 10µm **e** Quantitative analysis of nuclear/cellular MeCP2 fluorescence from z-projections. Mean ± SD from at least 30 individual cells (10 for WT MeCP2). Statistical analysis by two-way ANOVA with Bonferroni’s post hoc test (* *p* < 0.05, ** *p* < 0.01, *** *p* < 0.001). **f** Nuclear and cytoplasmic fractionation of 3xFLAG-MeCP2 p.S68X-expressing cells treated with G418 (100 ng/µl) and CC-885 (5 nM) as indicated, alongside MeCP2 WT controls (loaded at full and 10% amount). Nuclear and cytoplasmic fractions were run on the same gel to allow direct comparison. MeCP2 full-length and truncated p.S68X were detected using anti-FLAG antibody. Lamin A/C served as nuclear marker and actin served as a cytoplasmic mark to confirm fractionation identity. Lamin A/C cannot be detected in MeCP2-WT lanes due to overexposure of the previously detected Flag signal. Tubulin served as a loading control. N = 3. WT = Wildtype, UT = untransfected. Uncropped Western blots related to Fig. 4 are shown in Suppl. Figs. 3 and 4

Following the systematic dual-reporter screen across 14 clinically relevant *MECP2* PTCs, we selected p.S68X for full-length protein and localization studies to examine whether TR-mediated protein recovery restores nuclear targeting (Fig. 4a). The p.S68X variant was selected as a representative UGA stop codon not only due to its robust TR response in reporter assays, but also because this mutation results in the loss of the endogenous nuclear localization signal (NLS), thereby enabling functional assessment of nuclear targeting upon TR-mediated full-length restoration. Cells were left untreated or treated with G418 alone (40 ng/µL or 100 ng/µL) or in combination with CC-885 (1 nM or 5 nM) for 24 h. Concentrations of 40 ng/µL G418 and 5 nM CC-885 correspond to the experimentally defined application limit tolerated by HeLa cells based on viability measurements (Fig. 2).

Under untreated conditions, only truncated MeCP2 was detectable, consistent with dual reporter-based TR measurements (Fig. 4b, Suppl. Figure 3). Treatment with G418 alone induced low but measurable recovery of full-length MeCP2, reaching approximately 1% at 40 ng/µL and 4% at 100 ng/µL relative to WT controls (Fig. 4c). Co-treatment with CC-885 potentiated G418-mediated TR in a concentration-dependent manner. Notably, combination of 40 ng/µL G418 with CC-885 increased full-length recovery to approximately 4%, thereby reaching levels comparable to those achieved with 100 ng/µL G418 alone. At 100 ng/µL G418, CC-885 further enhanced TR to a maximum of 7%.

These results indicate that the potentiating effect observed in the reporter system is reflected at the protein level. Combined treatment with G418 and release factor degradation increased the recovery of full-length MeCP2 protein expression. To determine whether full-length recovered MeCP2 is associated with the correct subcellular localization, 3xFLAG-tagged MeCP2 p.S68X was further analyzed using confocal immunofluorescence microscopy. The p.S68X truncation results in a severely shortened protein lacking the downstream NLS, thereby preventing nuclear targeting that is readily observed for WT MeCP2 (Fig. 4d, Suppl. Figure 2). Conformingly, untreated cells expressing MeCP2 p.S68X displayed predominantly cytoplasmic fluorescence, with residual nuclear localization of approximately 20% (Fig. 4d, Suppl. Figure 2). As full-length recovery under untreated conditions was excluded by reporter assays and western blot analysis, this residual nuclear signal likely reflects passive diffusion of the truncated protein into the nucleus due to its reduced molecular size [30, 31].

Treatment with G418 partially restored nuclear localization to approximately 30% (Fig. 4d-e, Suppl. Figure 2), consistent with TR-mediated recovery of full-length MeCP2 that contains a functional NLS. Contrary to previous analyses, we did not observe a concentration-dependent effect by G418 treatment alone, possibly reflecting limited detection sensitivity at the protein level. Importantly, the combination with CC-885 resulted in a markedly stronger nuclear recovery of MeCP2. Using a combination of 100 ng/µL G418 with 5 nM CC-885 increased nuclear localization to over 40%. To further validate these findings, cytoplasmic and nuclear fractions of cells expressing MeCP2 p.S68X were analyzed by western blotting (Fig. 4f, Suppl. Figure 4). Cell fractionation and western blot detection were used to provide qualitative and supporting evidence for the nuclear localization of recovered full-length MeCP2. Fractionation purity was confirmed by the exclusive nuclear detection of Lamin A/C and the cytoplasmic restriction of beta-Actin. The Western blot detection of Flag signal and re-samling of the membrane with anti-Lamin A/C antibodies together with the overexposure of MeCP2-WT only allows Lamin A/C detection in MeCP2 p.S68X-transfected samples. Residual WT signal detected in cytoplasmic fractions likely reflects overexpression-related saturation of the nuclear import pathways (Fig. 4f, Supp. Figure 4). Notably, G418-mediated recovery of full-length MeCP2 was predominantly observed in nuclear fractions, with only trace amounts detectable in the cytoplasmic fraction, consistent with active nuclear import of the recovered full-length protein bearing a functional NLS. Co-treatment with CC-885 further increased nuclear full-length MeCP2 levels.

Together, these results demonstrate that aminoglycoside-induced TR at the p.S68X PTC recovers full-length MeCP2 protein and enables its nuclear localization. Co-treatment with CC-885 significantly enhances full-length MeCP2 and nuclear relocalization, supporting the concept that factor degradation potentiates the effect of TR inducers.

## Discussion

In this study, we show that eRF3a degradation by CC-885 and CC-90009 effectively potentiates aminoglycoside-mediated TR induction at a wide range of pathologically relevant *MeCP2* PTCs. eRF1 is responsible for stop codon recognition and peptidyl-tRNA hydrolysis, and eRF3a strongly stimulates both processes through its GTPase activity [32]. Thus, decreasing the intracellular eRF3a level increases the likelihood of near-cognate tRNAs incorporation at stop codons, in direct proportion to the residual eRF3a level. On their own, both eRF3a degraders did not induce TR, indicating their role as TR potentiators rather than TR inducers. Both, CC-885 and CC-90009, act as highly potent eRF3a degraders in the low nanomolar range, in line with previous reports [27, 33, 34], with CC-885 being more potent than CC-90009. CC-885 enhanced G418-mediated TR in a concentration-dependent fashion. Interestingly, TR efficiency continued to increase at CC-885 concentrations beyond 30 nM, at which point eRF3a protein was no longer detectable by Western blotting. This observation suggests that CC-885 may act through additional mechanisms beyond eRF3a degradation to promote TR. While the precise secondary mechanisms remain unknown, CC-885 is known to engage the cereblon E3 ligase complex broadly and may therefore affect additional substrates relevant to translational termination or ribosome dynamics. Further studies will be needed to investigate whether these effects are eRF3a-independent and whether they contribute meaningfully to the observed TR potentiation at higher compound concentrations.

Our results further underscore the strong dependence of TR efficiency on the SCC. It is well established that UGA stop codons are most responsive, whereas UAA codons show almost no response to aminoglycoside-induced TR [13, 20, 35–37]. Due to the TR-potentiating characteristics of CC-885, TR levels are strongly boosted at TR-permissive UGA stop codons (especially p.S68X, p.R198X, and p.R270X) to a maximum TR level of 40%, whereas UAA stop codons exhibited no detectable synergy. Increasing the CC-885 concentrations to toxic levels mediated TR at UAA stop codons, indicating that very low levels of eRF3a are sufficient to efficiently terminate translation at non-permissive stop codons.

We also tested the potential of CC-885 to boost gentamicin-mediated TR, a less toxic aminoglycoside compared to G418 [38–40]. Even when applied at concentrations ten-fold higher than G418, gentamicin alone induced only weak TR, including at PTCs that responded well to G418 treatment. In contrast, co-treatment with CC-885 strongly potentiated gentamicin-mediated readthrough, resulting in efficiencies comparable to those achieved with the highest tested G418 concentration. Yet, the potentiation was restricted to PTCs with high intrinsic inducibility (p.R198X, p.S68X), while no effects were detected at UAG or UAA PTCs. This further suggests that CC-885 functions primarily as a TR potentiator, rather than a universal TR inducer. While CC-885-mediated degradation of eRF3a reduces the efficiency of translation termination, it does not itself promote near-cognate tRNA incorporation at stop codons. Consequently, in the absence of an initial TR-inducing stimulus, such as aminoglycosides, eRF3a depletion alone is insufficient to trigger substantial TR. This mechanistic constraint explains why CC-885 enhances TR predominantly at PTCs that already exhibit measurable baseline responsiveness to aminoglycosides.

A particularly relevant finding is that the dual treatment allows a reduction of aminoglycoside concentration to 20% without compromising TR efficiency. The tolerable application limit even outperforms the TR inducibility of a 2.5-fold higher G418 concentration alone. Reducing the required aminoglycoside concentration to achieve a certain TR level while preventing cytotoxicity may provide a useful strategy in future studies. Of note, the application limit in this study was determined using HeLa cells and cannot be generalized, as a strong cell-type-dependent variability upon CC-885 treatment has been demonstrated recently [27]. Further, aminoglycosides such as gentamicin are associated with nephrotoxicity and ototoxicity, which limit their systemic clinical use. In contrast, G418 is not approved for therapeutic applications due to its pronounced cytotoxicity and is therefore primarily used as an experimental TR inducer in vitro. Additionally, the long-term physiological consequences of chronically reduced eRF3a levels remain unknown. The degradation of eRF3a constitutes a major disturbance of the translation termination machinery and potentially the proteome homeostasis. Furthermore, eRF3a has been shown to play a role in cell cycle progression beyond translation termination. eRF3a depletion induces G1 phase arrest through inhibition of the mTOR pathway, accompanied by a reduction in global translation rate [41]. CC-885-or CC-90009-induced eRF3a degradation may therefore affect cell proliferation and global protein synthesis in addition to its specific effect on translational readthrough, which represents a relevant consideration for the interpretation of future therapeutic applications of this approach. However, no diseases are associated with mutations in the *GSPT1* gene expressing eRF3a according to the Human Gene Mutation Database and here we used tolerable doses [42]. Nevertheless, other drugs with similar modes of action may provide safer alternatives. For example, ataluren, a clinically approved drug, was recently reported to inhibit release factor complex catalysis of translation termination [43]. As it may similarly function as a TR potentiator rather than a TR-inducing drug, its combination with TR inducers may allow a safer approach in reducing effective concentrations while maintaining cell survival. Indeed, it was recently demonstrated that ataluren, in combination with aminoglycosides yielded synergistic TR induction as observed by CC-885 treatment in this study [44, 45].

As the inserted amino acid at a PTC (W, C, R at UGA or Q, Y, K at UAA/UAG) might critically impair protein functionality, we also investigated the ability of recovered full-length MeCP2 to localize into the nucleus [10, 11, 46, 47]. Previously, it was shown that the nuclear import of MeCP2 relies on only one of two NLS sequences and that G418-induced TR recovers low amounts of MeCP2 p.S68X in the nucleus [3, 7, 48]. Here, we confirmed the nuclear recovery of the MeCP2 p.S68X mutant upon TR induction, indicating restoration of nuclear localization as a necessary prerequisite for MeCP2 function. Importantly, CC-885 treatment increased G418-mediated nuclear recovery of MeCP2, while simultaneously decreasing the cytoplasmic localization, indicating that the reduction in cytoplasmic MeCP2 p.S68X reflects its translocation to the nucleus, a finding validated by cell fractionation. Of note, immunofluorescence-based quantification of nuclear MeCP2 recovery cannot distinguish between full-length and truncated MeCP2, as both carry the FLAG tag. The truncated p.S68X protein (~ 16 kDa) falls below the size exclusion limit of the nuclear pore complex and may passively diffuse into the nucleus, contributing to the nuclear FLAG signal independently of TR [30, 31]. This passive nuclear accumulation likely accounts for the elevated baseline nuclear signal observed in untreated cells and may lead to a systematic overestimation of TR efficiency by immunofluorescence. Further, nuclear re-localization does not fully guarantee functionality of recovered full-length MeCP2, and further studies may evaluate readthrough-recovered MeCP2 activity at chromatin and in disease-relevant neuronal models.

Taken together, our findings demonstrate that eRF3a degradation is an effective strategy to potentiate aminoglycoside-mediated TR induction. Combination treatments allow the reduction of required aminoglycoside concentrations on the one hand and potentiation of weak TR-inducing drugs on the other hand. However, this approach is limited to TR accessible PTCs due to the potentiating characteristics of eRF3a degradation. Little is known, however, about the physiological consequences of eRF3a degradation, and this approach raises serious concerns about potential side effects. Therefore, further work is required to clarify and improve safety and tissue-specific efficacy.

## Material and methods

### Dual reporter and full-length constructs

The following plasmids were used in this study:

**Table:** 

Plasmid number (PST)	Plasmid name	Source
1596	pcDNA3.1(+)_RFP_GFP	Schilff et al. (2021)
1600	PST1596_Rett-pR198X	Lebeda et al. (2024)
1601	PST1596_Rett-pS49X	Lebeda et al. (2024)
1602	PST1596_Rett-pQ406X	Lebeda et al. (2024)
1739	PST1596_Rett-pR168X	Lebeda et al. (2024)
1740	PST1596_Rett-pR270X	Lebeda et al. (2024)
1741	PST1596_Rett-pY141X	Lebeda et al. (2024)
1742	PST1596_Rett-pQ170X	Lebeda et al. (2024)
1743	PST1596_Rett-pR255X	Lebeda et al. (2024)
1744	PST1596_Rett-pS204X	Lebeda et al. (2024)
1745	PST1596_Rett-pQ244X	Lebeda et al. (2024)
1746	PST1596_Rett-pK175X	Lebeda et al. (2024)
1747	PST1596_Rett-pS68X	Lebeda et al. (2024)
1748	PST1596_Rett-pK177X	Lebeda et al. (2024)
1749	PST1596_Rett-pK363X	Lebeda et al. (2024)
1810	Rett WT	Brendel et al. (2009)
1819	Rett c.203C > G p.S68X	Lebeda et al. (2024)

### Cell culture

HeLa cells were cultured in Dulbecco’s Modified Eagle Medium (DMEM) supplemented with 1% glutamine, 1% penicillin/streptomycin, and 10% fetal calf serum (FCS) at 37 °C, 5% CO_2,_ and 90% humidity. Cells were seeded at defined densities depending on the assay: 3 × 10^5^ cells/well in 6-well plates for immunoblotting, 1 × 10^5^ cells/well in 12-well plates for immunofluorescence, 3 × 10^4^ cells/well in 96-well plates for dual reporter assays, and 5 × 10^5^ cells/100 mm dish for cell fractionation.

For immunofluorescence, cells were transfected with 400 ng plasmid DNA using Effectene (Qiagen). For all other experiments, cells were transfected with polyethylenimine Max (PEI, MW 40,000; Polysciences; used at a ratio of 3:1 PEI:DNA). DNA amounts were 150 ng/well for 96-well plates, 2 µg/well for 6-well plates and 9 µg for 100 mm cell culture dishes. Transfection reagent was removed no later than 16 h after transfection. For TR induction, transfection medium was replaced with fresh medium containing G418 (Carl Roth), supplied as aqueous solution, and CC-885 or CC-90009 (both MedChemExpress), dissolved in DMSO at stock concentrations of 1 µM and 30 µM, respectively. Cells were incubated for 24 h prior to analysis.

### Translational readthrough dual reporter assay

The principles of the dual reporter assay and TR quantification were described before [49]. Cells were washed with 150 µL PBS, detached with 35 µL of 0.5% trypsin (Sigma) for seven minutes, and resuspended in 165 µL phenol red-free DMEM supplemented with 1% penicillin/streptomycin and 10% FCS. 96-well plate flow cytometry on the Guava EasyCyte (Cytek) was performed using 488 nm and 532 nm lasers. HeLa cells were gated by forward scatter (23,000 to 75,000) and side scatter (12,500 to 67,500). Gated cells with an RFP signal > 750 or a GFP signal > 100 were included in the following TR calculations. Background GFP signal was subtracted from all samples using an RFP-expressing construct (PST1880). TR was calculated as the GFP to RFP ratio normalized to the 100% TR control vector (PST1596). For each measurement, PST1596 and untransfected HeLa cells were included. Gating and calculations were performed using RStudio (R Core Team).

### Cell viability assay

Cell viability was assessed using the OrangU Cell Counting Reagent (Cell Guidance Systems), a WST-8-based colorimetric assay measuring mitochondrial dehydrogenase activity as a proxy for cell viability. On day 1, a standard curve was generated by seeding 0–20,000 HeLa cells per well in a 96-well plate in triplicate. Cells were allowed to attach for 2 h at 37 °C and 5% CO₂, after which 10 µl of OrangU solution was added per well and incubated for 3 h. Absorbance was measured at 450 nm using a microplate reader. For viability assessment under treatment conditions, 2,000 cells per well were seeded into a 96-well plate and incubated for 2 h for cell adhesion. Afterward, cells were treated with G418 (0, 20, 40, 60, 80, or 100 ng/µl) and CC-885 (0, 1, 5, or 30 nM) in all pairwise combinations and incubated for another 24 h at 37 °C and 5% CO₂. Following treatment, 10 µl of OrangU solution was added per well and incubated for exactly 3 h at 37 °C and 5% CO₂. Absorbance was measured at 450 nm, and cell numbers were calculated from the standard curve.

### Cell fractionation

Cells were washed once and harvested in 1 mL ice-cold PBS followed by centrifugation at 4 °C, 6000xg for 5 min. The supernatant was removed, and the cell pellet was resuspended in 100 µL CE buffer (10mM HEPES pH 7.9, 10 mM KCl, 0.1 mM EDTA, 0.3% NP40 (fresh), 1 × protease inhibitor (fresh)) and incubated on ice for 5 min with vortexing every minute. After centrifugation at 3000xg for 5 min at 4 °C, the supernatant (cytoplasmic fraction) was transferred into a new tube. The pellet was washed twice with 100 µL of CE buffer lacking Nonidet P-40 and centrifuged at 3000xg for 5 min at 4°C. The pellet was resuspended in 40 µL of NE buffer (20 mM HEPES pH 7.9, 0.4 M NaCl, 1 mM EDTA, 25% glycerol, 1 × protease inhibitor (fresh) and incubated on ice for 10 min with vortexing every minute. Samples were centrifuged at 14,000xg for 5 min at 4 °C, and the supernatant (nuclear fraction) was transferred into a new tube.

### Cell lysis and immunoblotting

Cells were washed with 1 mL of ice-cold PBS, collected in ice-cold PBS, and centrifuged for 5 min at 16,000 xg. The supernatant was removed, and cells were lysed with 100 µL lysis buffer (20 mM Tris, 150 mM NaCl, 0.1% Tween-20, 0.5% Triton X-100, 1X proteinase inhibitor Complete, pH 7.6 (Thermo)). Samples were incubated on ice for 15 min, sonicated for 20 s at 40% amplitude (Branson), and centrifuged at 16,000 xg for 15 min at 4°C. Protein concentration measured by a BCA assay (Thermo).

60 µg protein per sample was loaded onto an SDS gradient gel (4% - 20%). Proteins were transferred to PVDF membrane (MeCP2 p.S68X) or nitrocellulose (all other samples) using the Trans-blot Turbo Transfer system (Bio-Rad) using standard settings. Membranes were blocked for 30 min at RT with 5% milk powder in TBS-T (20 mM Tris, 150 mM NaCl, 0.1% Tween-20, pH 7.6) and probed overnight at 4 °C with the primary antibodies: anti-Flag M2 (Merck F1804, 1:000), anti-Tubulin (CellSignalling 3873S, 1:1000 only for cytoplasmic and nuclear fractions), anti-Lamin A/C (CellSignalling 4C11, 1:500 only for cytoplasmic and nuclear fractions), anti beta-Actin (Invitrogen MA5-16,410, 1:1000 only for cytoplasmic and nuclear fractions) and anti-GAPDH (Sigma G9545, 1:1000). After three wash steps with TBS-T, membranes were incubated for 1 h at RT with HRP-conjugated secondary antibodies (Jackson Immuno-Research Laboratories, 1:10,000): donkey anti-mouse IgG (715–035–150) or goat anti-rabbit IgG (111–035-003). Bands were visualized using ECL substrate (Bio-Rad) and recorded using the Chemostar Touch Imager (Intas). Densitometric analyses were performed in Fiji-ImageJ. Readthrough efficiency was calculated as the ratio of full-length to total (full-length and truncated) MeCP2 band intensity.

### Immunofluorescence and microscopy

Transfected cells were seeded on 18 mm coverslips (12-well plate), washed with sterile PBS and fixed with 4% paraformaldehyde (Thermo) for 10 min at RT. After three washes with PBS, cells were permeabilized with 0.5% TritonX-100 in PBS for 5 min and blocked with 5% BSA for 30 min at RT. Coverslips were incubated overnight at 4 °C with primary anti-Flag M2 antibody (Merck F1804, 1:500 in PBS with 1% BSA). After three washes with PBS, coverslips were incubated for 1 h at RT with Alexa Fluor 647-conjugated secondary goat anti-mouse antibody (Invitrogen A32728, 1:1000 in PBS), DAPI (Roth, 0.1 μg/mL) and Alexa Fluor 488 phalloidin (Invitrogen, 1:400 in PBS). Coverslips were mounted (Dako) and left to dry overnight at RT. An LSM900 confocal fluorescence microscope (Zeiss) with a 63 × oil immersion objective (NA 1.40) and GaAsP-PMT detectors was used for imaging. Image processing and quantifications were performed in Fiji-ImageJ.

### Statistical data analysis

All statistical analyses were performed in GraphPad Prism. Details of specific tests, significance thresholds, and multiple-comparison corrections are reported in the respective figure legends.

## Data Availability

The data that support the findings of this study are detailed in the supplementary material. Further data are available from the corresponding author upon reasonable request.
